# SCRAPPER Regulates the Thresholds of Long-Term Potentiation/Depression, the Bidirectional Synaptic Plasticity in Hippocampal CA3-CA1 Synapses

**DOI:** 10.1155/2012/352829

**Published:** 2012-12-20

**Authors:** Hiroshi Takagi, Mitsutoshi Setou, Seiji Ito, Ikuko Yao

**Affiliations:** ^1^Cellular & Molecular Synaptic Function Unit, Initial Research Project, Okinawa Institute of Science and Technology Promotion Corporation, 1919-1, Tancha, Onna 904-0495, Japan; ^2^Molecular Gerontology Research Group, Mitsubishi Kagaku Institute of Life Sciences (MITILS), 11 Minamiooya, Machida 194-8511, Japan; ^3^Department of Molecular Anatomy, Hamamatsu University School of Medicine, 1-20-1 Handayama, Higashi-ku, Hamamatsu 431-3192, Japan; ^4^Department of Medical Chemistry, Kansai Medical University, 10-15 Fumizono-cho, Moriguchi 570-8506, Japan; ^5^Precursory Research for Embryonic Science and Technology (PRESTO), Japan Science and Technology Agency (JST), 7 Goban-cho, Chiyoda-ku 102-0076, Japan

## Abstract

SCRAPPER, which is an F-box protein encoded by *FBXL20*, regulates the frequency of the miniature excitatory synaptic current through the ubiquitination of Rab3-interacting molecule 1. Here, we recorded the induction of long-term potentiation/depression (LTP/LTD) in CA3-CA1 synapses in E3 ubiquitin ligase SCRAPPER-deficient hippocampal slices. Compared to wild-type mice, *Scrapper*-knockout mice exhibited LTDs with smaller magnitudes after induction with low-frequency stimulation and LTPs with larger magnitudes after induction with tetanus stimulation. These findings suggest that SCRAPPER regulates the threshold of bidirectional synaptic plasticity and, therefore, metaplasticity.

## 1. Introduction 

It is not clear how the activities of synaptic proteins are regulated under physiological conditions. Activity-induced decreases and increases in synaptic efficacy can occur in mammalian neurons. Modulation of the activity of synaptic protein complexes is important for the control of synaptic efficacy. We previously reported that these modulatory processes involve protein degradation [1], as well as control at the level of transcription [2], translation [3], and translocation [4–6]. In the central nervous system (CNS), the strength of synaptic efficacy can undergo robust use-dependent changes. Synaptic efficacy can be long-term potentiated (LTP) either by high-frequency tetanic stimulation [7] or by low-frequency stimulation (LFS) that is paired with large depolarization of the postsynaptic membrane [8]. In contrast, long-term depression (LTD) of synaptic strength can be induced by prolonged LFS [9] or by LFS that is paired with relatively small depolarizations of the postsynaptic membrane [10, 11].

 The ability of CNS synapses to undergo such bidirectional synaptic plasticity has long been considered the cellular mechanism for information storage in the brain [12–15]. The sliding threshold of bidirectional synaptic plasticity is an example of metaplasticity or the plasticity of synaptic plasticity. The mechanisms underlying the sliding threshold of bidirectional synaptic plasticity in the CNS have been extensively examined, with particular focus on the intrinsic properties of the glutamatergic postsynapse. For example, it is now well known that the induction of both LTP and LTD in glutamatergic synapses requires the activation of the *N*-methyl-**d**-aspartate (NMDA) subtype of postsynaptic glutamate receptors [9, 16–23]. The sliding of the threshold of bidirectional synaptic plasticity is regulated by the composition of the NMDA receptor subunits (NR2A, NR2B) [24, 25]. In contrast, the mechanisms underlying the sliding threshold of bidirectional synaptic plasticity in glutamatergic presynapses are not clear. Furthermore, it has been reported that the frequencies of miniature excitatory synaptic currents (mEPSC) correlate with the degree of LTD in the CA1 region of hippocampal slices [26]. Thus, the continual synaptic activity of spontaneous mEPSCs is thought to correlate with the induction of LTP/LTD. 

 Activity-dependent synaptic plasticity is associated with synaptic protein turnover that involves the ubiquitin proteasome system (UPS). It has been shown that changes in synaptic transmission can regulate the levels of synaptic proteins through the UPS [27–31]. However, the specific spatiotemporal dynamics of degradation of proteins, especially presynaptic proteins, during the formation of synaptic plasticity remains to be clarified. Previously, we reported that SCRAPPER, which is an F-box protein encoded by *FBXL20*, regulates the frequency of mEPSCs through the ubiquitination of the Rab3-interacting molecule 1 (RIM1) [1, 32]. RIM1, which has been reported to determine the density of Ca^2+^ channels and vesicles at the presynaptic active zone, is known as a synaptic vesicle priming factor [33, 34]. Using *Scrapper*-knockout (SCR-KO) mice, we showed that SCRAPPER acts as a component of an E3 ubiquitin ligase and that it regulates the frequency of mEPSCs at hippocampal CA3-CA1 synapses through the presynaptic degradation of RIM1 [1]. Furthermore, we reported histopathological abnormalities and neuronal degeneration in many brain regions (e.g., hippocampus, corpus striatum, cerebral cortex, and cerebellum) [35] and behavioral abnormalities (e.g., impairment of performance contextual but not cued fear conditioning tests, which implies a loss of function in the hippocampus) [36] in the SCR-KO mouse. These findings indicate that SCRAPPER can regulate higher order brain function through the presynaptic degradation of RIM1. In this study, we tested the contribution of SCRAPPER to the sliding threshold of bidirectional synaptic plasticity formation in CA1 synapses with SCR-KO mice. 

## 2. Methods

### 2.1. Animals

SCR-KO mice have been described previously [1]. Because of the lethality of SCR-KO mice that are genetically backcrossed to the C57/BL6J line, the analysis of SCR-KO mice was performed on littermates that were derived from mating heterozygous mutant mice with a hybrid 129Sv/C57BL6 background. Mice were housed in a room with a 12 h light/dark cycle with *ad libitum* access to food and water. Animal care and experiments were conducted in accordance with the general policies of the Mitsubishi Kagaku Institute of Life Sciences and Kansai Medical University.

### 2.2. Western Blot Analysis

 A rabbit anti-SCRAPPER antibody was raised against amino residues 321–380 of mouse SCRAPPER that was expressed in bacteria, as previously described [1]. Western blotting was performed by conventional methods [1, 37]. 

### 2.3. Preparation of Hippocampal Slices for Electrophysiology

The techniques used in this experiment were essentially identical to those described previously [1, 37]. Hippocampal slices were prepared from 3- to 4-week-old wild-type (WT) and SCR-KO mice. Mice were decapitated, and the brain was rapidly removed and placed in ice-cold high-sucrose solution containing 250 mM sucrose, 3.5 mM KCl, 26 mM NaHCO_3_, 10 mM **d**-glucose, 1.3 mM NaH_2_PO_4_, 1.2 mM MgSO_4_, and 3.8 mM MgCl_2_. Transverse 300 *μ*m thick slices were prepared with a microslicer (PRO7, Dosaka EM Co., Ltd., Kyoto, Japan) and then incubated for at least 120 min at room temperature in a normal solution (NS) containing 125 mM NaCl, 3.5 mM KCl, 26 mM NaHCO_3_, 10 mM **d**-glucose, 1.3 mM NaH_2_PO_4_, 1.2 mM MgSO_4_, 3.8 mM MgCl_2_, and 2.5 mM CaCl_2_. For recording, 300 *μ*m thick slices of mouse hippocampus were maintained at 30°C in a recording chamber that was perfused with a modified NS containing 124 mM NaCl, 3.5 mM KCl, 26 mM NaHCO_3_, 1.3 mM NaH_2_PO_4_, 1.2 mM MgSO_4_, 2 mM CaCl_2_, and 10 mM **d**-glucose. All solutions were saturated with a 95% O_2_/5% CO_2_ gas mixture throughout the experiments. 

### 2.4. Electrophysiological Recordings of Hippocampal Slices

 Field excitatory postsynaptic potentials (fEPSPs) were recorded either from the outer dendritic regions of CA1 while stimulating at the same level in the stratum radiatum or close to the pyramidal cells. Stimulation was performed with monopolar glass electrodes, and recordings were obtained using artificial cerebrospinal fluid-filled glass pipettes (resistance < 2 MΩ). Test stimuli consisted of biphasic 100 *μ*s pulses of constant current that were delivered by stimulus isolation units. Basal synaptic transmission was monitored by alternating LFS (every 60 s). Baseline responses had to be stable for at least 30 min before these stimuli were delivered. The signals were amplified with an amplifier (EPC-7, HEKA Elektronik Dr. Schulze GmbH, Lambrecht/Pfalz, Germany). fEPSPs were digitized (1 kHz) with the pClamp6 acquisition system, stored on magnetic media, and analyzed off-line with the Clampfit 8.0 analysis system (Molecular Devices, LLC, Sunnyvale, CA, USA). The strength and duration of the stimulus pulse were adjusted to elicit a population spike at the cell body layer with an amplitude of 40–60% of the maximum spike amplitude. After checking the stability of the responses to a test stimulus that was given every 60 s, a train of LFS (900 pulses at 1 Hz) was delivered in order to elicit LTD. A tetanus pulse (100 pulses at 100 Hz) was delivered to elicit LTP. After delivery of the LFS and tetanus, the test stimuli were repeated every 60 s, and responses were recorded for at least 60 min. 

### 2.5. Statistical Analysis

 The changes in the slopes of the excitatory postsynaptic potentials (EPSPs) before and after stimulation are expressed as the mean ratio of the control level. All values are expressed as the mean ± standard error of the mean (SEM), and the results were analyzed for statistical significance (*P* < 0.05) with Student's *t*-tests (two tailed). 

## 3. Results

### 3.1. Smaller Magnitudes of LTD Were Induced by LFS in SCR-KO Mice

**Figure 1:**
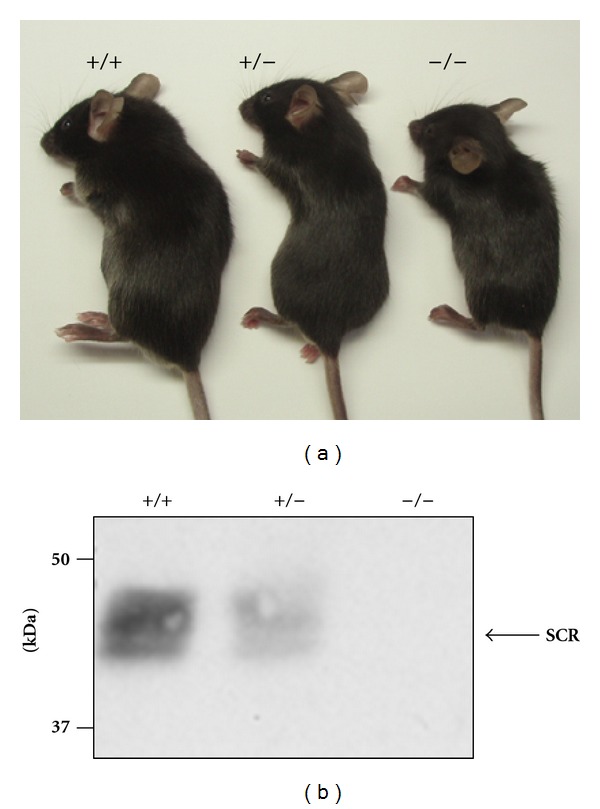
Inhibition of SCRAPPER protein expression in *Scrapper*-knockout (SCR-KO) mice. (a) Appearance of SCR-KO mice (Yao et al. [1], with permission from Elsevier). (b) Western blot analysis of SCRAPPER in wild-type (WT) or SCR-KO mouse hippocampus. Five microgram of protein in the hippocampal homogenate was applied to each lane and immunoblotted by the anti-SCRAPPER antibody. The SCRAPPER signal was not observed for SCR-KO mice. +/+, WT; +/−, heterozygote; −/−, homozygote SCR-KO mice.

**Figure 2:**
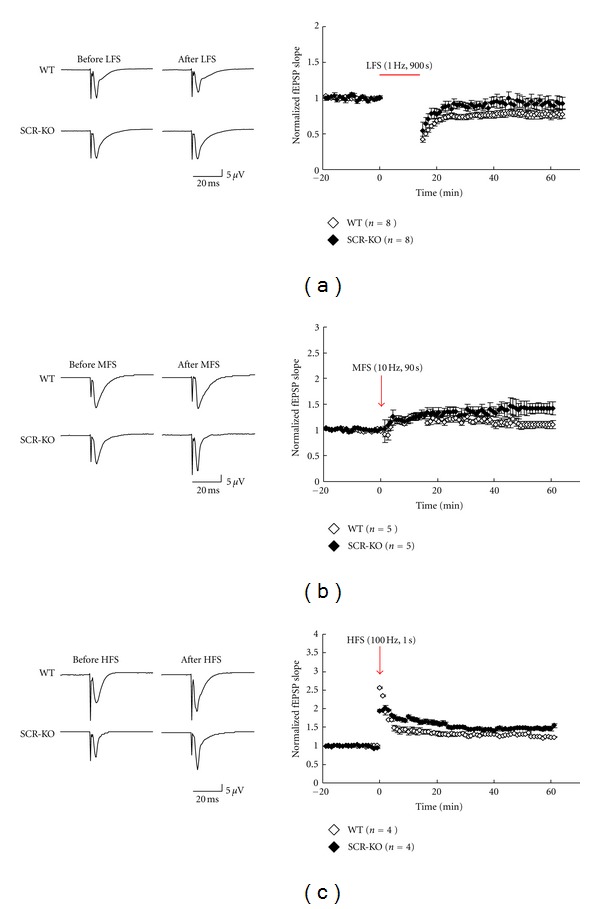
Long-term potentiation/depression (LTP/LTD) induction by stimulation at different frequencies in wild-type (WT) or *Scrapper*-knockout (SCR-KO) hippocampal slices. Left column: records of representative experiments in which LTP/LTD was induced. Low-frequency stimulation (LFS, 1 Hz, 900 s) (a), middle-frequency stimulation (MFS, 10 Hz, 90 s) (b), or high-frequency stimulation (HFS, 100 Hz, 1 s) (c) was used as the input. Right column: normalized means (± standard error of the mean (SEM)) of field excitatory postsynaptic potential (fEPSP) slopes from WT or SCR-KO hippocampal slices. Each point represents a single measure of the initial slope of the population excitatory postsynaptic potentials (EPSPs) evoked by stimulation of the Schaffer collaterals at 1/60 Hz. The horizontal bar or the arrow represents the period of stimulation. The number of mice, *n* = 8, 5, and 4 in (a), (b), and (c), respectively. (*◊*, WT; ◆, SCR-KO).

 SCR-KO mice had smaller body sizes than their WT littermates did (Figure 1(a)), and they did not express the SCRAPPER protein (Figure 1(b)). We recorded LTP/LTD induction in hippocampal CA3-CA1 synapses in brain slices of SCR-KO mice. First, we induced extended periods of LFS in the range of 1 Hz in order to yield an LTD. The lasting consequences of 900 pulses that were delivered at 1 Hz are illustrated in Figure 2(a). The responses were often facilitated immediately after the onset of the conditioning stimulation, but during conditioning, the response magnitude always progressively declined to values below the initial baseline (data not shown). When the baseline measurements were resumed (at 1/60 Hz), the first response was always depressed and was even below the value attained during LFS (Figure 2(a)). Although there was usually some recovery in the response magnitude over the next several minutes, the population fEPSP slope always reached a plateau at a value that was significantly depressed compared to the value for the pre-LFS control period (Figure 2(a)).

 The mean magnitudes of the LTDs in the fEPSPs measured at 45–55 min after the end of LFS were 0.8 ± 0.05 (WT; *n* = 8) and 0.92 ± 0.03 (SCR-KO; *n* = 7) of the pre-LFS levels. The value that was measured 45–55 min after the end of the LFS was significantly higher (*P* < 0.05) in SCR-KO mice than in WT mice. 

### 3.2. Larger Magnitudes of LTP Were Induced by Tetanus Stimulation in SCR-KO Mice

As illustrated in Figure 2(b), 900 pulses at 10 Hz (middle frequency stimulation) resulted in weak LTP in WT mice (1.13 ± 0.05 of the control at 45–55 min after stimulation; *n* = 5). In contrast, the same number of pulses at 10 Hz produced, on average, significantly larger changes in SCR-KO mice (1.39 ± 0.06 of the control at 45–55 min after stimulation; *n* = 6) (*P* < 0.05).

 As shown in Figure 2(c), tetanic stimulation (100 Hz for 1 s) produced LTP in WT mice (1.26 ± 0.05 of control; *n* = 4). The magnitude of the LTPs in SCR-KO mice (1.47 ± 0.05 of control; *n* = 4) was significantly larger than that of WT mice. Namely, smaller magnitudes of LTDs were induced by LFS in SCR-KO mice. However, larger magnitudes of LTP were induced by tetanus stimulation in SCR-KO mice. 

### 3.3. The Threshold Was Shifted in the SCR-KO Mice

**Figure 3:**
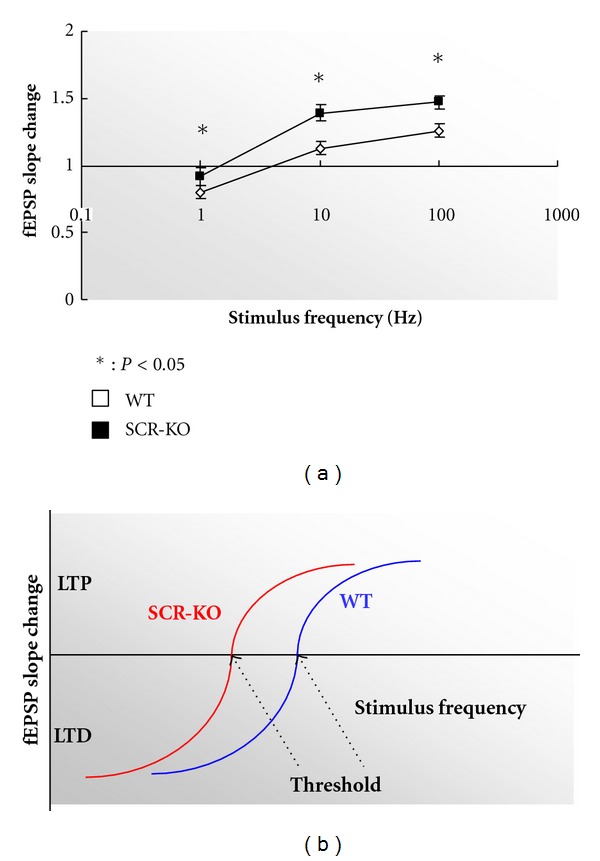
Comparison of the field excitatory postsynaptic potential (fEPSP) slope changes between wild-type (WT) and *Scrapper*-knockout (SCR-KO) mice at different stimulation frequencies. (a) Means of 10 consecutive sweeps before and after (45–55 min) stimulation. Mean (± SEM) effect of the stimulus of the conditioning stimulation delivered at various frequencies on the response measured 50 min after conditioning (□, WT; ■, SCR-KO) *P < 0.05 (*t*-test). (b) Schematic model of the long-term potentiation/depression (LTP/LTD) threshold changes in SCR-KO hippocampus and the dependence on stimulus frequency.

 Prior synaptic or cellular activity influences the degree or threshold of the subsequent induction of synaptic plasticity, which is a process known as metaplasticity. Next, we compared the threshold of bidirectional synaptic plasticity between the WT and SCR-KO mice. As shown in Figure 3(a), the threshold of bidirectional synaptic plasticity was shifted to the lower frequency area. The schematic model of the LTP/LTD threshold in Figure 3(b) illustrates that the shifts to the lower frequency area in SCR-KO hippocampus depended on stimulus frequency. 

## 4. Discussion

### 4.1. Altered Threshold of Bidirectional Synaptic Plasticity in SCR-KO Hippocampus

Various studies of synaptic plasticity in the CNS have provided insights into the cellular and molecular mechanisms underlying certain types of learning and memory. As shown in Figure 2, the reduction in the threshold of the bidirectional synaptic plasticity at hippocampal CA1 synapses was observed in the SCR-KO than in WT mice. Our results indicated that SCRAPPER regulated the threshold of bidirectional synaptic plasticity. 

The effects of altering the threshold in the present study were explained by the different values of the postsynaptic responses (perhaps, the integrated postsynaptic depolarization or intracellular Ca^2+^ concentration levels) through the increased mEPSC frequencies during the conditioning stimulation. Our results demonstrated the lowering of the threshold of bidirectional synaptic plasticity (metaplasticity) by presynaptic modulation by the ubiquitin ligase SCRAPPER. Because it is likely that some mechanisms regulate the expression or activation of SCRAPPER through the cAMP pathway [1], it is possible that the cAMP-dependent pathway and the stimulation of the pathway regulate not only late-phase LTP, but also bidirectional synaptic plasticity [38]. Unfortunately, the mechanisms of SCRAPPER activation in physiological conditions are still unknown. Future studies are needed to clarify the activation mechanisms of SCRAPPER, and knowledge of these mechanisms will help in elucidating the physiological implications of SCRAPPER-dependent bidirectional synaptic plasticity. 

### 4.2. Regulation of mEPSC Frequencies by the Modification of RIM1 with SAD Kinase and Another E3 Ligase K-Spot

In our previous study, SAD kinase upregulated the frequency of mEPSCs through RIM1 phosphorylation [39, 40]. In addition, SAD kinase has been shown to be required for the polarization and integration of neurites in the transmission of information in the brain [41–43]. Thus, such a multikinase pathway that links extracellular signals to the intracellular machinery could be connected to RIM1 phosphorylation and alterations of the frequencies of mEPSCs. Like SCRAPPER, K-spot, which is a component of the E3 ligase for Munc-13, has also been shown to regulate the frequency of mEPSCs [44]. Together, these results indicate that SAD kinase and K-spot regulate the threshold of bidirectional synaptic plasticity in hippocampal CA3-CA1 synapses. Furthermore, SCRAPPER is expressed not only in the hippocampus, but also ubiquitously throughout the whole brain [1]. These findings suggest that SCRAPPER can regulate the threshold of bidirectional synaptic plasticity through the degradation of RIM1 in other brain regions. These regulatory mechanisms may be involved in the formation of contextual fear memory, which is largely suppressed in SCR-KO mice [36]. 

### 4.3. Detection of Alterations in Molecular Architecture in the Brain of SCR-KO Mice

A functional defect in SCRAPPER leads to neurotransmission abnormalities, which result in neurodegenerative phenotypes [35]. SCRAPPER has been reported to be ubiquitously expressed in many brain regions [1]. The pathological abnormalities and neuronal degeneration in the brain of SCR-KO mice [35, 36] suggest that SCRAPPER may play a crucial and fundamental role in the maintenance of the developmental changes in synaptic plasticity through the regulation of the threshold of bidirectional synaptic plasticity. 

 To our knowledge, this is the first report suggesting that E3 ubiquitin ligase (SCRAPPER) can regulate hippocampal CA3-CA1 synapses through the modulation of neurotransmitter release. Recently, our group succeeded in visualizing the *in situ *two-dimensional anatomy of neurotransmitter release in brain slice preparations with imaging mass spectrometry (IMS) analysis [45]. IMS is useful for the visualization of the distribution of various biomolecules [35, 46–48]. This approach can be useful in clarifying the fundamental role of SCRAPPER in the maintenance of the threshold of synaptic plasticity from the standpoint of activity dependency, brain regionality, and development. Future studies are needed to reveal how SCRAPPER regulates higher brain function through the modulation of the threshold of bidirectional synaptic plasticity under physiological conditions (*in vivo*).

## 5. Conclusions

 The balance of protein synthesis and UPS-dependent degradation is important for the maintenance of bidirectional synaptic plasticity. With E3 ubiquitin ligase SCRAPPER-deficient hippocampal slices, we recorded LTP/LTD induction in CA3-CA1 synapses. Compared to WT mice, SCR-KO mice showed smaller magnitudes of LTDs after LFS and larger magnitudes of LTPs after tetanus stimulation. These findings suggest that SCRAPPER regulates the threshold of bidirectional synaptic plasticity, that is, the metaplasticity of hippocampal CA3-CA1 synapses through the regulation of mEPSC frequency through RIM1 degradation.
